# Prevalence of factors contributing to unplanned hospital readmission of older medical patients when assessed by patients, their significant others and healthcare professionals: a cross-sectional survey

**DOI:** 10.1007/s41999-023-00799-6

**Published:** 2023-05-24

**Authors:** Lisa Fønss Rasmussen, Louise Grode, Ishay Barat, Merete Gregersen

**Affiliations:** 1 Department of Research, Horsens Regional Hospital, Sundvej 30, 8700 Horsens, Denmark; 2 Department of Medicine, Horsens Regional Hospital, Sundvej 30, 8700 Horsens, Denmark; 3 Department of Geriatrics, Aarhus University Hospital, Palle Juul Jensens Boulevard 99, 8200 Aarhus N, Denmark

**Keywords:** Patient readmission, Causes of readmission, Hospitalisation, Surveys and questionnaires, Aged, Cross-sectional studies

## Abstract

**Objective:**

To describe the prevalence of factors contributing to readmission of older medical patients perceived by patients, significant others and healthcare professionals and to examine the agreement of factors contributing to readmission.

**Methods:**

This cross-sectional survey was conducted at Horsens Regional Hospital from September 2020 to June 2021. Patients aged ≥ 65 years and who were readmitted within 30 days were included. The questionnaire covered eight themes: disease; diagnosing, treatment and care; network; organisation; communication; skills and knowledge; resources; and practical arrangements. Response groups were patients, significant others, GPs, district nurses and hospital physicians. Outcomes were the prevalence of factors contributing to 30-day readmission and inter-rater agreement between respondents.

**Results:**

In total, 165 patients, 147 significant others, 115 GPs, 75 district nurses and 165 hospital physicians were included. The patients’ median age was 79 years (IQR 74–85), and 44% were women. The following were the most prevalent contributing factors: (1) relapse of the condition that caused the index admission, (2) the patient could not manage the symptoms or illness, (3) worsening of other illnesses or conditions, (4) the patient was not fully treated at the time of discharge and (5) the patient’s situation was too complex for the medical practice to handle. Kappas ranged from 0.0142 to 0.2421 for patient—significant other dyads and 0.0032 to 0.2459 for GP—hospital physician dyads.

**Conclusion:**

From the perspectives of the included respondents, factors associated with the disease and its management were the most prevalent contributors to readmission for older medical patients. Agreement on the contributing factors was generally low.

**Trial registration:**

Clinical trial number NCT05116644. Registration date October 27, 2021.

**Supplementary Information:**

The online version contains supplementary material available at 10.1007/s41999-023-00799-6.

## Key summary points

**Aim:**

To describe the prevalence of perceived factors contributing to the unplanned hospital readmission of medical patients aged 65 years and above.

**Findings:**

Perceived factors contributing to unplanned readmission relate to the patient’s illness and how it is managed. Patients and significant others as well as general practitioners and hospital physicians have low inter-rater agreement and different views on the contributing factors.

**Message:**

General practitioners and significant others find it challenging to meet the needs, demands and expectations of patients who are discharged hastily and have not recovered.

**Supplementary Information:**

The online version contains supplementary material available at 10.1007/s41999-023-00799-6.

## Introduction

Approximately 20% of all hospitalised patients aged over 65 years are readmitted within the first 30 days after discharge [1]. Readmissions negatively affect patients, significant others, healthcare systems and health finances [2–4].

Risk factors and predictors for the readmission of older medical patients have been extensively examined in recent decades. Co-morbidity and functional disability prior to hospital admission, length of hospital stay, increasing age, male gender, living in a nursing home, method of referral, discharge destination, low socioeconomic status and frailty [5–9] are known risk factors. Additionally, self-reported symptoms, such as shortness of breath, anxiety, depression and fatigue, are predictors for hospital readmission [10]. Organisational factors, such as problems in the transition from hospital to home [11], poor quality of care in the primary setting [12, 13], insufficient discharge planning and insufficient communication between hospital and primary care professionals, have also been associated with readmission [14, 15].

Despite this knowledge, readmission rates are still high. To address this challenge, we need to examine readmissions differently. One way to do this is to investigate the factors contributing to older medical patients’ readmission. Studies examining contributing factors are primarily qualitative and have examined younger patient groups from merely one or two perspectives [16–20].

To our knowledge, no studies have examined the contributing factors amongst older medical patients from multiple perspectives. We expect that a multi-perspective approach would provide exceptional knowledge that could lead to the prevention of unplanned readmissions. Therefore, the aims of this study are to (1) describe the prevalence of factors contributing to unplanned hospital readmission for medical patients aged 65 years and above within 30 days of discharge and (2) examine the inter-rater agreement between patients and significant others and between general practitioners (GP) and hospital physicians of the perceived factors contributing to readmission.

## Methods

### Design and setting

This cross-sectional survey was conducted in Denmark at Horsens Regional Hospital (HRH) and four surrounding municipalities (Odder, Hedensted, Skanderborg and Horsens) from September 2020 to June 2021.

HRH is a public teaching hospital in the Central Denmark Region with 240 beds that had 21,677 acute hospital admissions in 2020. The medical department is divided into three sub-specialised wards: cardiology, internal medicine/respiratory medicine/gastroenterology (MW1) and internal medicine/endocrinology/geriatric medicine (MW2). This study was conducted in MW1 (30 beds) and MW2 (19 beds). The readmission rate amongst older medical patients at HRH was 20.2% in 2019 and 20.6% in 2021.

The total number of inhabitants in the four municipalities in 2020 was 223,210. Of these, 43,149 (19.3%) were aged ≥ 65 years, with 53% women. Odder Municipality accounted for 5366 (23.5%) inhabitants aged ≥ 65 years, Hedensted 9536 (20.4%), Skanderborg 11,410 (18.2%) and Horsens 16,837 (18.5%) [21].

This study was reported in accordance with (1) the Strengthening the Reporting of Observational Studies in Epidemiology (STROBE) checklist for cross-sectional studies [22] and (2) a Consensus-Based Checklist for Reporting of Survey Studies (CROSS) [23].

### Participants

The questionnaire’s five response groups consisted of readmitted patients and their significant others, GPs, district nurses and hospital physicians. The inclusion process is elaborated below:

- Patients: Patients were eligible for inclusion if they were (1) aged ≥ 65 years, (2) acutely readmitted to MW1 or MW2 within 30 days after discharge from index admission at MW1 or MW2 and (3) living in one of the four municipalities.
  Patients were excluded if (1) they did not speak or understand Danish, (2) they were declared terminally ill or (3) the readmission was planned.
  Eligible patients were identified daily from an automatically generated report from the Business Intelligence Portal in Central Denmark Region. Patients who met the inclusion criteria were consecutively recruited. If possible, readmitted patients were approached and enrolled in the study within 72 h after readmission.
- Significant others: Significant others—if possible, a son or daughter of the patient—were included in the study. Input and experience from the development and pilot testing of the questionnaire revealed that a son/daughter would have a broader insight into contributing factors relating to the eight themes, as compared to an older spouse. If it was not possible to recruit a son or daughter, a spouse or close friend was included as the patient’s significant other. Significant others were contacted by phone or in person to introduce them to the study and obtain their e-mail address.
- GPs: Danish citizens have a personal GP in the area of their residence. The questionnaire was sent directly to the patients’ GPs, who were paid a fee for completing the questionnaire according to their collective agreement.
- District nurses: The district nurses from the municipality of Horsens did not participate due to lack of human resources and vacant positions; thus, these respondents were excluded a priori.
  The questionnaire was sent a priori to a chosen administrative employee in each of the home healthcare systems of the three participating municipalities (Odder Hedensted and Skanderborg). This person identified the district nurse with the most knowledge and insight about each patient’s condition. All identified district nurses were given time to complete the questionnaire during working hours.
- Hospital physicians: The first senior hospital physicians to attend to the patients after readmission were identified through electronic patient records. A speciality registrar was identified if a senior hospital physician had not attended to the patient within the first 2 days.

All GPs, district nurses in the municipalities and hospital physicians in the medical wards were potential survey respondents. Therefore, they received information on the survey by e-mail, oral presentations or in members’ journals before the study started to ensure a high response rate. The collaborators were encouraged to distribute the information to their colleagues and employees. Before the study began, representatives from the participating municipalities and GPs were asked if they had any requests regarding the questionnaire delivery. Hence, the distribution was tailored according to the respondents’ needs.

### Outcomes

The primary outcome was the prevalence of factors contributing to 30-day readmission at a group level. The secondary outcome was the agreement between patients and significant others and between hospital physicians and GPs at a patient level.

Group level was defined as unpaired pooled data from each response group regardless of patient relation. Patient level was defined as data paired and analysed for each readmitted patient and his/hers significant other, hospital physician and GP. They all assessed factors contributing to the same patient’s readmission.

### Data sources

Besides responses from the survey, data were retrieved from the CROSS-TRACKS Cohort [24] to describe the patient population in detail.

#### Questionnaire

A formative questionnaire was developed prior to the study. Details on the development and validation process are described in ‘Development and validation of a questionnaire identifying contributing factors to readmission amongst older medical patients’ by Rasmussen et al. (unpublished). The questionnaire was based on (1) semi-structured interviews with five patients, five significant others, five GPs, five district nurses and five hospital physicians (see online resource ESM_1 for the questions asked) and (2) existing evidence on risk factors and predictors for readmission by older medical patients. It contained 49 items and eight free-text sections. Response categories were in the form of a 5-point Likert scale (strongly disagree, disagree, partly agree, agree and strongly agree), multiple choice, not relevant and don’t know.

Questionnaire items were grouped into the following eight themes: (1) disease; (2) diagnostics, treatment and care; (3) network; (4) organisation; (5) communication; (6) skills and knowledge; (7) resources; and (8) practical arrangements. The items were formulated as statements, such as ‘relapse of the condition that caused the index admission’.

The questionnaire was modified into a shorter version for patients with only yes/no/don’t know response categories. It contained only 41 statements within the same eight themes and eight free-text sections. See online resource ESM_1 for details about the questionnaires. Respondents were not obliged to respond to the statements.

#### Questionnaire administration

Questionnaire administration and data collection were performed by the researcher (LFR), a nurse with 6 years of experience or a trained nursing student. All had been comprehensively trained in patient enrolment, data collection and questionnaire administration prior to the start of the study.

Following enrolment, the questions and response categories were read aloud to the patient, and their responses were entered digitally into a REDCap database [31]. If the patient was too ill to answer, they were revisited up to four times. If the patient was still too ill to participate, they were registered as a non-responder.

All respondents received a personal unique questionnaire link to prevent multiple responses. Questionnaires were sent electronically to the significant others, GPs, district nurses (if patients received home care or nursing) and hospital physicians. On rare occasions, the questionnaires were completed over the phone if a significant other did not have an e-mail account or experienced other response barriers. GPs were asked if they had been in contact (telephone, video or personal consultation) with the patient between discharge from index admission and readmission. If no, the questionnaire was terminated without entering data; if yes, the GPs were asked to complete the questionnaire. Patients normally took 20–45 min to complete the questionnaire, whereas healthcare professionals generally needed 20–30 min.

When the surveys were completed, the responses were automatically relocated to and stored in the REDCap database. Following agreement with the collaborators from each response group, survey reminders were sent out electronically once a week, with a maximum of five reminders. If the questionnaires were not returned due to the abovementioned circumstances, they were reported as ‘not included’. If respondents received the questionnaire but did not complete it, they would appear as ‘non-respondents’.

### Study size

Based on local historical data from HRH in 2019, we estimated that it would be possible to include 250–300 patients over a 1-year period.

### Data analysis and statistical methods

Descriptive analysis was used to characterise the population of readmitted patients and to describe the prevalence of contributing factors. We focussed on factors contributing to readmission. Thus, we only included highly agree, agree and yes responses in the analysis. These categories were pooled into a binary variable called ‘agree with the statement’, meaning that this factor contributed to the readmission. Hence, ‘not relevant’, ‘don’t know’ and disagree responses were not included. Missing data were not reported because they were not essential for addressing the study’s aims.

The ten most prevalent contributing factors perceived by each response group were merged into a bar chart illustrating the most contributing factors perceived by all five response groups.

The factors with the highest prevalence were analysed using Cohen’s kappa (*κ*) to examine the inter-rater agreement between (1) patients and their significant others and (2) GPs and hospital physicians at the patient level. To describe the strength of agreement, we applied the definition described by Landis and Koch [25]. The agreement was considered poor if *κ* < 0.00, slight if *κ* = 0.00–0.20, fair if *κ* = 0.21–0.40, moderate if *κ* = 0.41–0.60, substantial if *κ* = 0.61–0.80 and almost perfect if *κ* = 0.81–1.00.

### Patient and public involvement

Managers and mid-level managers from MW1 and MW2 wards at HRH, managers from the home healthcare systems in Odder, Hedensted and Skanderborg municipalities and a GP working partly at the HRH and partly in medical practice participated in the planning of the survey, consecutive evaluations of the questionnaire distribution and response barriers as well as other practical aspects.

### Ethics approval

Approval was obtained from the Danish Data Protection Agency (case no. 1-16-02-113-19). According to the Danish Scientific Ethical Committees Act section 14, subsection 2, approval by the Central Denmark Region Ethical Committee was not required. Informed consent was obtained from the participants, who could withdraw their consent at any time. All of the included patients provided informed consent. Approval to contact and collect data from the significant other, GP, district nurse and hospital physician was also obtained through the informed consent. Significant others provided informed consent on behalf of patients who were not mentally or physically capable of consenting. Despite consenting on behalf of the patient, the significant other only responded to the questionnaire on their own behalf, hence with their own subjective assessment of the contributing factors. If the patient was not capable of consenting and completing the questionnaire, the questionnaire was left blank and registered as non-respondents. To link the questionnaire responses at the patient level, each patient’s personal identification number was used as an identifier for their questionnaire. These numbers were then replaced by a non-personal ID number, thus making the data pseudonymous.

The survey responses did not affect the treatment of the patients. This study was performed in accordance with the 1964 Declaration of Helsinki and its later amendments [26]. All correspondences containing personally identifiable data were encrypted to comply with the General Data Protection Regulation. Questionnaire links were sent by secure e-mail or through the electronic patient record system. E-mails to the significant others were sent through REDCap. These e-mails did not contain personally identifiable data.

## Results

### Participants

**Fig. 1:**
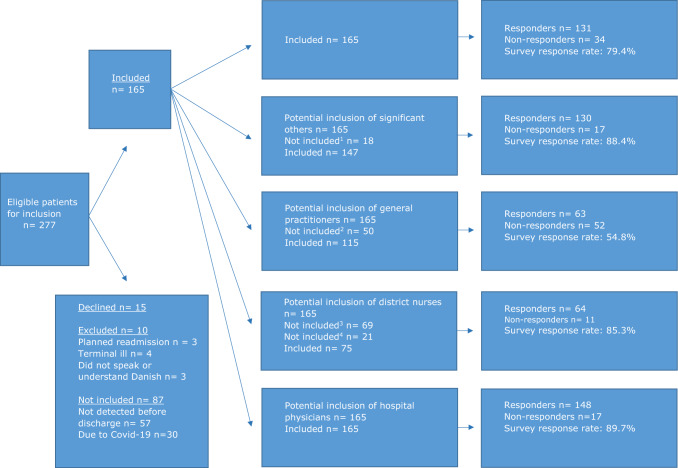
Flow diagram. Inclusion process and response rates for patients, significant others, general practitioners, district nurses and hospital physicians. 1: Declined to participate, 2: the GP did not have contact with the patient between discharge and readmission, 3: Horsens municipality did not participate due to a lack of human resources, 4: no need for home care services

During the study period, 277 readmitted patients were eligible for inclusion. Of those, 15 declined to participate and ten were excluded. In addition, 87 were not included mainly due to (1) quick admission and discharge during weekends and public holidays, when project workers were not present and (2) the second wave of the COVID-19 pandemic. Hence, 165 patients were included. See Fig. 1 for more details.

The patients’ median age was 79 years (74–85 years), and 44% were women (Table 1).

**Table 1:** Baseline characteristics

	Readmitted patients
Total, *n* (%)	165 (100)
Gender, *n* (%)	
Female	73 (44.2)
Male	92 (55.8)
Age, median (IQR)	79 (74–85)
Age groups, *n* (%)	
65–69	26 (15.8)
70–74	26 (15.8)
75–79	37 (22.4)
80–84	36 (21.8)
85–89	27 (16.4)
≥ 90	13 (7.8)
Municipality, *n* (%)	
Odder	20 (12.1)
Hedensted	36 (21.8)
Skanderborg	31 (18.8)
Horsens	78 (47.3)
LOS in days during index admission, median (IQR)	4 (3–7)
Housing, *n* (%)	
Own home	148 (89.7)
Nursing home	17 (10.3)
Civil status, *n* (%)	
Married	71 (43.0)
Divorced	22 (13.4)
Not married	6 (3.6)
Widowed	61 (37.0)
Unknown	5 (3.0)
Social status, *n* (%)	
Cohabiting	118 (71.5)
Living alone	47 (28.5)
CCI, *n* (%)	
No co-morbidity	35 (21.2)
Low	36 (21.8)
Moderate	25 (15.2)
High	69 (41.8)
Leading cause of index admission, *n* (%)	
Pneumonia	30 (18.2)
Other infections	25 (15.2)
COPD	18 (10.9)
Urinary tract infections	10 (6.1)
Other endocrine/malnutrition	8 (4.9)
Cardiovascular disease	7 (4.2)
Dementia or delirium	5 (3.0)
Dehydration	3 (1.8)
Abnormal blood sample values at discharge, *n* (%)	
CRP (> 8.0 mg/l)	142 (86.1)
eGRF (< 60 ml/min)	98 (59.4)
Haemoglobin	125 (75.8)
Female: (< 7.3|> 9.5 mmol/l)	
Male: (< 8.3|> 10.5 mmol/l)	
Sodium (< 137|> 145 mmol/l)	48 (29.1)
Leukocyte (< 3.5|> 10.0 × 10/l^9^)	69 (41.8)
Potassium (< 3.5|> 4.6 mmol/l)	31 (18.8)
Albumin	155 (93.9)
40–70 years: (< 36|> 45 g/l)	
70–105 years: (< 34|> 45 g/l)	
Abnormal vital signs at discharge, *n* (%)	
Systolic blood pressure (< 100|> 200 mmHg)	5 (3.0)
Pulse rate (< 50|> 90 bpm)	27 (16.4)
Saturation (< 93 SpO_2_%)	45 (27.3)
Respiratory rate (< 10|> 16 breaths/min)	96 (58.2)
Visits to the GP within 30 days after discharge, *n* (%)	57 (34.6)
Visits to the out-of-hour doctor within 30 days after discharge, *n* (%)	22 (13.3)
Polypharmacy, *n* (%)	160 (97.0)
Admissions 1 year prior to index admission, *n* (%)	131 (20.6)

LOS: length of stay, CCI: Charlton Co-Morbidity Index (scores calculated based on the International Classification of Diseases-10 diagnostic codes of 19 conditions [39, 40]), COPD: chronic obstructive pulmonary disease, CRP: C-reactive protein, eGRF: estimated glomerular filtration rate, GP: general practitioner, polypharmacy: ≥ 5 daily drugs [41] and index admission: the admission prior to the readmission. Abnormal vital signs are defined using the Early Waring Score for hospitalised adults in Denmark [42]. Normal blood values are defined using the List of Analysis from Central Denmark Region [43]

Overall, the survey response rates at the group level were 79.4% by the patients, 88.4% by the significant others, 54.8% by the GPs, 85.3% by the district nurses, and 89.7% by the hospital physicians (Fig. 1). At the patient level, all five response groups completed the questionnaire in 16 patient cases (9.7%). For additional detail on the respondents’ completion of the questionnaires, see online resource ESM_2.

### Contributing factors

**Fig. 2:**
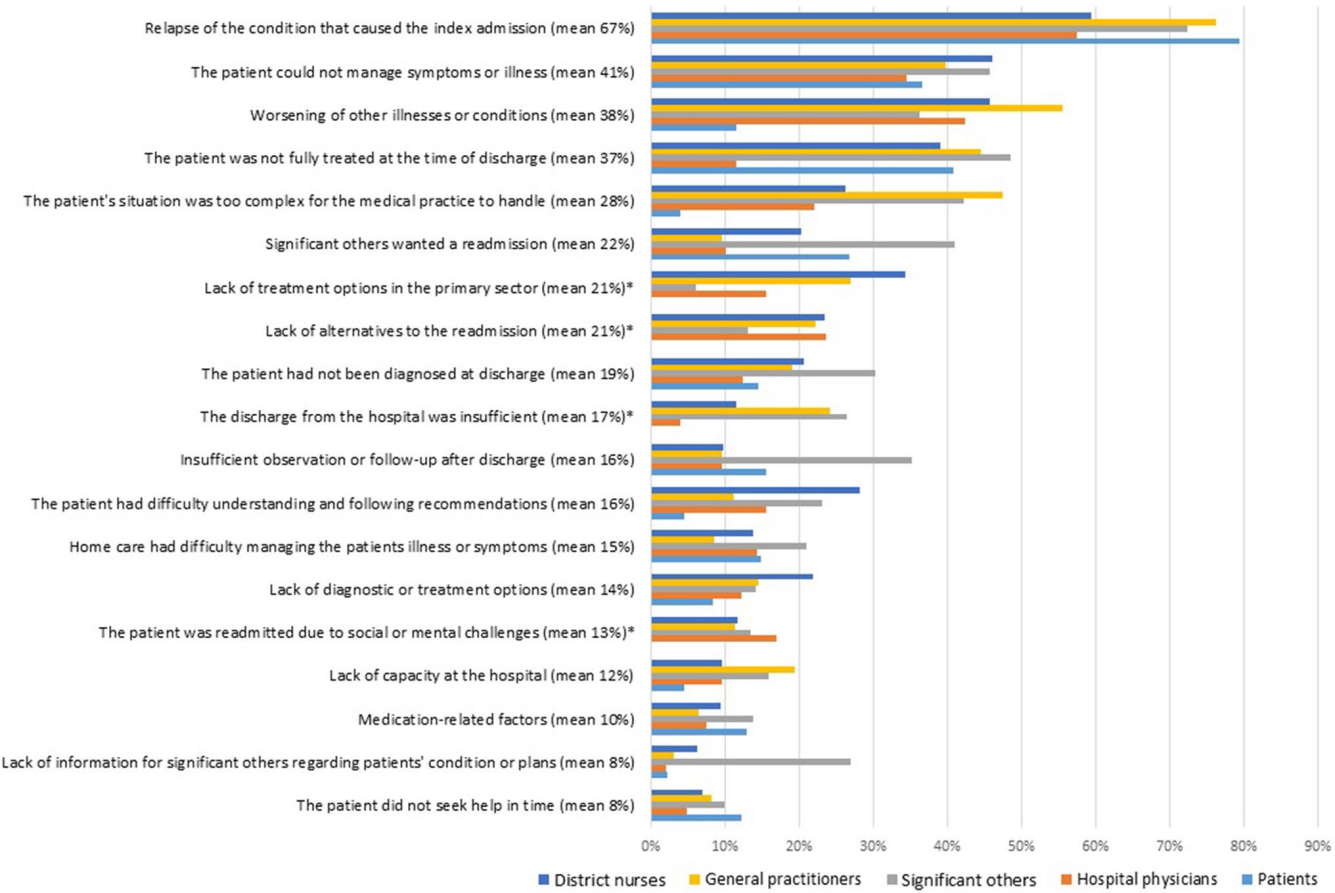
Bar chart of the factors contributing most. The chart illustrates the 19 factors contributing most to readmission seen from the perspectives of patients, significant others, general practitioners, district nurses and hospital physicians. The factors are listed according to their mean value. *The statement was not included in the patient questionnaire

The ten most prevalent contributing factors perceived by each of the five response groups were merged into one bar chart to compare their responses, see online resource ESM_3 for frequencies of statement responses. To a great extent, the five groups perceived the same factors to be the most contributing. Thus, merging those factors resulted in the 19 most prevalent contributing factors (Fig. 2).

Overall, the factors with the highest prevalence across groups were “relapse of the condition that caused the index admission” (mean: 67%, range: 57–79%), “the patient could not manage the symptoms or illness” (41%, 37–46%), “worsening of other illnesses or conditions” (38%, 11–56%), “the patient was not fully treated at the time of discharge from index admission” (37%, 11–48%) and “the patient’s situation was too complex for the patient’s medical practice to handle” (28%, 4–47%).

The contributing factors with lowest prevalence across the five groups in Fig. 2 were “medication-related factors” (10%, 6–14%), “lack of information for significant others regarding patients’ condition or plans” (8%, 2–27%) and “the patient did not seek help in time” (8%, 7–12%).

The patients had noticeably lower prevalence than the other groups in “worsening of other illness or conditions” and “the patient’s situation was too complex for the medical practice to handle”.

The significant others had a considerably higher prevalence, and thus differed from the other response groups, in “lack of information for significant others regarding patients’ condition or plan”, “insufficient observation or follow-up after discharge”, “the patient had not been diagnosed at discharge” and “significant others wanted a readmission”.

Compared with the four other groups, the hospital physicians less frequently perceived “the discharge from the hospital was insufficient” and “the patient was not fully treated at the time of discharge” to be factors contributing to readmission.

#### Factors perceived in the hospital and primary healthcare

**Fig. 3:**
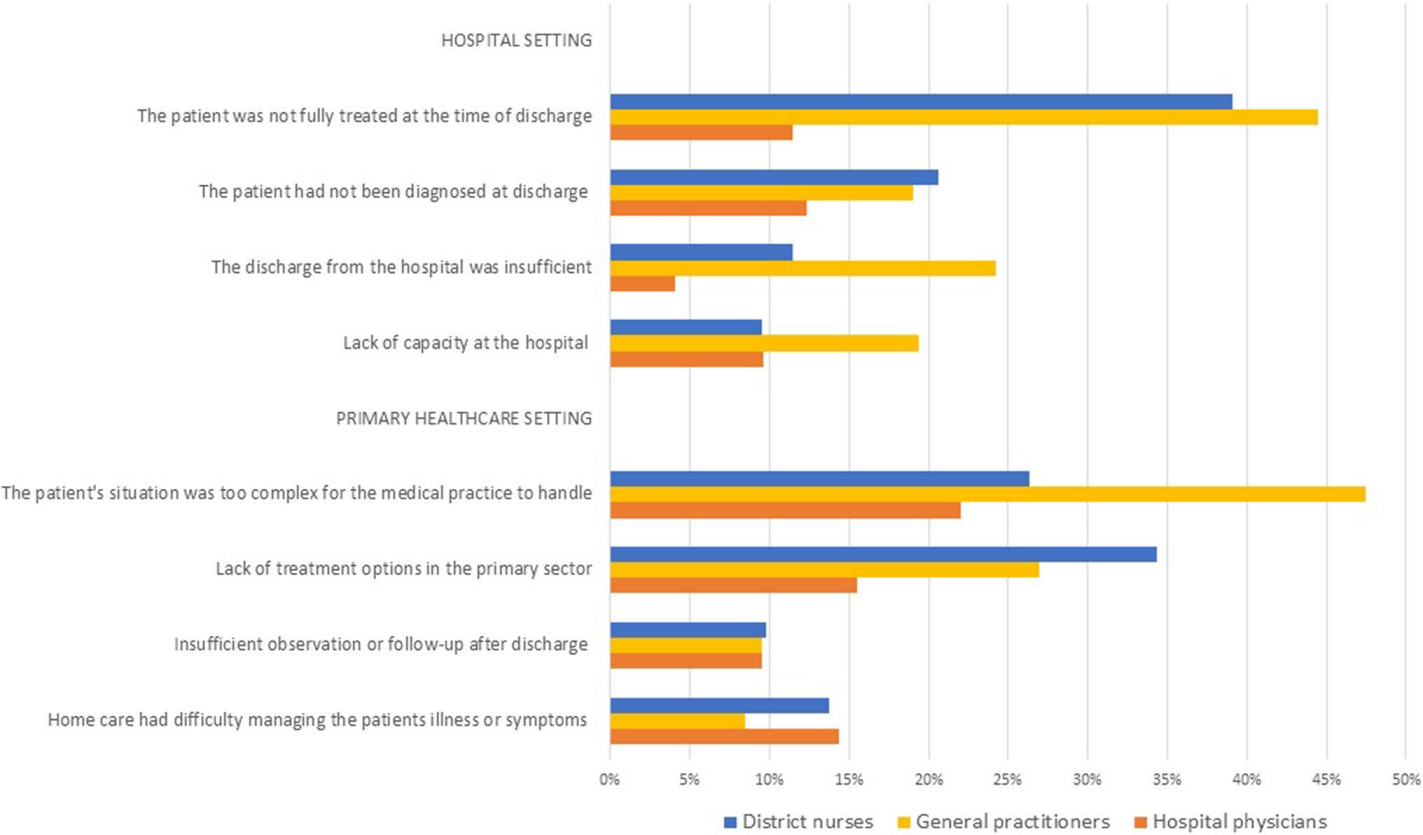
Bar chart of the contributing factors occurring in hospital and primary healthcare settings. The chart illustrates the contributing factors occurring in the hospital and primary setting seen from the perspectives of general practitioners, district nurses and hospital physicians

Figure 3 provides a comparison of the healthcare professionals’ perceptions of factors that occurred in the hospital and primary care. To a great extent, the GPs’ and district nurses’ responses were similar. They perceived “the discharge from the hospital was insufficient”, “the patient was not fully treated at the time of discharge from index admission” and “the patient had not been diagnosed at the time of hospital discharge” to be contributors to readmission.

Compared with the GPs and district nurses, the hospital physicians had lower prevalence in the following factors: “the patient was not fully treated at the time of discharge”, “the discharge from the hospital was insufficient” and “lack of treatment options in the primary sector”.

### Inter-rater agreement at the patient level

The inter-rater agreement was calculated once both the patient and their significant other had responded to the statement. The same was applicable for the patient’s GP and hospital physician.

#### Patients and significant others

Table 2 shows that the inter-rater agreement of factors perceived by patients and their significant others were all slight, except for one, which was fair. The only factor for which the level of agreement between the significant other and the patient was better than what could be expected by chance was “the patient was not fully treated at the time of discharge from index admission” (95% CI 0.06; 0.43).

**Table 2:** Inter-rater agreement between patients and significant others and at the patient level

The five most contributing factors	Inter-rater agreement between patients and significant others	
	Agreement, % (95% CI)	Kappa, *κ* (95% CI)
Relapse of the condition that caused the index admission (*n* = 100)	63.0 (0.53; 0.72)	0.0294 (− 0.17; 0.23)
Worsening of other illnesses or conditions (*n* = 63)	68.3 (0.55; 0.79)	0.0816 (− 0.14; 0.31)
The patient was not fully treated at the time of discharge from index admission (*n* = 99)	62.6 (52.3; 72.1)	0.2421 (0.06; 0.43)*
The patient could not manage the symptoms or illness (*n* = 100)	47.0 (0.37; 0.57)	− 0.0711 (− 0.26; 0.12)
The patient’s situation was too complex for the patient’s medical practice to handle (*n* = 44)	56,8 (0.41; 0.72)	0,0142 (− 0.13; 0.16)

*Statistically significant

To illustrate the interpretation of Tables 2 and 3, it can be stated that the patients and significant others both answered the statement “relapse of the condition that caused the index admission” in 100 cases. They agreed with the patient in their perception of the contributing factors in 63.0% of cases. This corresponds to a kappa of 0.0294. The agreement was not larger than what could be expected by chance, as it was not statistically significant. However, the agreement was statistically significant in “the patient was not fully treated at the time of discharge from index admission”, thereby anticipating that the agreement was not caused by chance.

**Table 3:** Inter-rater agreement between the general practitioners and hospital physicians at the patient level

The five most contributing factors	Inter-rater agreement between GPs and hospital physicians	
	Agreement	Kappa
	% (95% CI)	*κ* (95% CI)
Relapse of the condition that caused the index admission (*n* = 59)	55.9	0.1112
	(42.7; 68.8)	(− 0.112; 0.334)
Worsening of other illnesses or conditions (*n* = 50)	50.0	0.0032
	(35.5; 64.5)	(− 0.272; 0.278)
The patient was not fully treated at the time of discharge from index admission (*n* = 59)	61.0	0.1019
	(47.4; 73.5)	(− 0.055; 0.259)
The patient could not manage the symptoms or illness (*n* = 59)	64.4	0.2459
	(50.9; 76.4)	(− 0.006; 0.498)
The patient’s situation was too complex for the patient’s medical practice to handle (*n* = 50)	58.0	0.16
	(43.2; 71.8)	(− 0.095; 0.415)

#### GPs and hospital physicians

Table 3 shows that agreement between the GPs and hospital physicians was slight, except for one that was fair, and none was statistically significant. Again, the agreement was not larger than what could be expected by chance.

#### Response time

The patients completed the questionnaire during the readmission; thus, they had the shortest response time of all the response groups. GPs and hospital physicians generally had the longest response time (online resource ESM_4).

## Discussion

According to the patients, significant others, GPs, district nurses and hospital physicians, out of 49 factors, the five factors contributing most to readmission were “relapse of the condition that caused the index admission”, “the patient could not manage the symptoms or illness”, “worsening of other illnesses or conditions”, “the patient was not fully treated at the time of discharge from index admission” and “the patient’s situation was too complex for the patient’s medical practice to handle”.

The level of agreement between the patients and their significant others as well as the GPs and hospital physicians was low.

It is difficult to directly compare and discuss the present results with previous research due to this being a state-of-the-art study. To our knowledge, studies assessing contributing factors were either qualitative or only assessed contributing factors from one, two or three perspectives. That said, the results from studies with some similarities to ours are presented. In line with our findings, these studies found that relapse of symptoms or disease, worsening of other illnesses or conditions [18, 27–29], premature discharge and insufficient post-discharge follow-up [17, 30, 31], insufficient discharge [2], lack of ability to self-manage the symptoms and disease [16], low discharge readiness [32] and untreated health problems [33] contributed to readmission. Also, in line with our results, one study found low kappa statistics ranging from 0.02 to 0.34 for patient—physician dyads and 0.03 to 0.68 for patient—caregiver dyads [20]. Another study found a low inter-rater agreement, with kappa ranging from 0.02 to 0.30 for physician dyads [19].

The five factors with the highest prevalence were related to the symptoms and diseases and how patients and GPs found it difficult to handle and manage those. This indicates that the patients were not sufficiently treated and were being discharged prematurely from the hospital. This, however, is a well-documented fact [34].

The patients’ responses were related to their illness, self-management and treatment, and they found it difficult to manage their symptoms and/or illness at home. This may be related to insufficient post-discharge follow-up, not being fully treated at the time of discharge and not feeling ready to be discharged. The healthcare system must thoroughly consider how to address these factors to improve self-management amongst older ill patients to manage their conditions successfully and to prevent readmission.

In the significant others’ perception, it seems that the primary and secondary healthcare systems could not offer sufficient treatment, care or follow-up to their readmitted family members nor deliver sufficient information to the significant others. This indicates that the significant others did not feel fully capable of assisting and supporting the patients. However, this is not surprising, as they experience the entire trajectory across sectors and institutions and thus identify errors, absences and incoherent trajectories. It is, however, crucial for the healthcare system to address this, as treatment and care tasks are increasingly delegated to significant others. It is also known that caregiver involvement in transitions reduces hospital readmission [35] which underlines the need to address these problems. This is supported by Kongensgaard et al., who found the attendance of a significant other during geriatric team home visits to be associated with lower unplanned 30-day readmission amongst severely frail patients living alone [36].

The highly specialised doctors and nurses in hospitals hand over the treatment and care responsibilities to GPs and district nurses, who are primarily generalists. Our results suggest that in GPs’ perception, these complex and sick patients are difficult to treat within the existing healthcare setting and with the treatment and diagnostic options and resources available.

It seems as hospital physicians generally assess hospital-based factors as less contributing to readmission compared to GP’s and district nurses. In contrast, GP’s and district nurses often assess factors in the primary health care setting as contributing factors. Hence, it seems as hospital physicians are more likely to use a defence mechanism when answering the questionnaire resulting in projection of the responsibility to other parts of the healthcare system.

Inter-rater agreement between the patient and the significant other as well as the GP and the hospital physician for individual patients was poor. This suggests that doctors attending to and treating the same patient have different opinions and perspectives on why the patient had been readmitted. These diverging views may be attributable to the fact that one is a generalist and the other is a specialist or because they represent different healthcare sectors with different structures, insights, resources and services.

The question is whether it is realistic to achieve a high level of agreement when respondents represent different sectors, levels of severity or acuteness and specialties. This leads a discussion on the patient-centred trend. Based on our findings, a patient-centred approach is complex and challenging in the real world. Older medical patients may have limited energy and insight, and significant others may be frustrated, anxious and have high expectations of the healthcare system. In addition, healthcare professionals have different views. It is assumed to be challenging to agree on expectations and goals when the perceptions are diverging. Therefore, to actually deliver patient-centred treatment with a high level of consensus amongst patients, significant others and healthcare professionals call for more (1) insight, knowledge and understanding of the entire patient trajectory across sectors, available resources and the different healthcare institutions and professions, (2) involvement, insight and collaboration with the patient and significant others and (3) collaboration between healthcare professionals across sectors.

Our study has various strengths. We examined the contributing factors from five different perspectives, which has contributed with unique knowledge on which further actions and studies can be built. Additionally, we included a generally older medical population, which widely represents patients in teaching hospitals, thereby increasing the generalisability. Furthermore, it must be assumed that the questionnaires were exhaustive, as no new contributing factors were identified through the free-text sections. The questionnaire content validity was good, as it was developed based on qualitative interviews with several representatives from all five response groups, thereby increasing the internal validity of the study results. Compared with another study [19], the GP and hospital physician response rates were remarkably high in this study. For the GPs, it was 55% in this study versus only 36% in that by Herzig et al. For hospital physicians, it was 90% versus 74%. This indicates a well-prepared study.

Our study has some limitations. First, we only have responses from all five perspectives in 16 patient cases. This results in less insight on contributing factors on patient level. However, it was not possible to statistically compare all five perspectives on patient level using Kappa statistics, which makes the limited number of complete dataset less problematic.

Perspectives on contributing factors assessed by the district nurses were not compared with other response groups’ perspectives on patient level. Their perspectives were not directly comparable to the GPs’ and hospital physicians’ perspectives due to different educational background and tasks. This limitation may be problematic, however, we argue that excluding this group from the kappa analysis contribute to more valid results.

We assumed that the most valid responses on contributing factors would be obtained from those with insight and knowledge on the patient trajectory in the time from the discharge from the index admission until the readmission. Therefore, we did not collect data from GPs who had not been in contact with the patient in the period as well as district nurses if patients were self-sufficient. This may also have led to the limited number of complete data sets on patient levels. However, we argue that the results of this study are more valid, than if we had included all possible respondents.

Not systematically including spouses may also be a limitation as they are often living with the patients and thus having a detailed knowledge and understanding of the disease, situation and contributing factors to readmission. However, we chose to include sons and daughters before spouses. This decision was made after feedback from the questionnaire development and pilot tests as they revealed that a son/daughter had a broader insight into contributing factors as compared to an older spouse. Including spouses before sons and daughters would probably have given the same result or less diverse perspectives on contributing factors.

Selection bias may be present, as the most severely ill or oldest patients may have been too ill or fatigued to participate [37]. This may have had a minor effect on the validity. Unfortunately, we could not assess whether the non-participant group differed significantly from the included patients by comparing their baseline characteristics. In addition, we expected to include 250–300 patients, yet only 165 participated. The lower inclusion rate was due to the COVID-19 pandemic, as fewer older patients were being (re)admitted, and several of those hospitalised had been diagnosed with COVID-19, compared with the pre-study period. The results might have been slightly different if we had included more patients.

End-aversion bias is also a threat when using a Likert scale [38]. However, we were not focussing on the response nuances and thus pooled all agree and yes responses.

Recall bias is also a threat to validity. Healthcare professionals comprised the groups with the longest response time. Hospital physicians attend to several new patients daily, and their contact typically has a short duration. This may affect recall of a specific situation. In contrast, GPs and district nurses generally have regular contact with a patient over a long time period, so recalling the last contact may be more straightforward. However, they were all encouraged to read their notes on the patient’s record to refresh their memory before completing the questionnaire. This may have minimised the recall bias. Lastly, district nurses from Horsens Municipality did not participate due to lack of human resources. Horsens is a much larger city than Odder, Hedensted and Skanderborg, thus patients from Horsens may have different characteristics. Obtained data and the prevalence of contributing factors may have been different if district nurse from Horsens had been included. This may affect the internal validity and generalisability.

This study examined the prevalence and agreement of factors contributing to unplanned readmissions amongst medical patients aged 65 years and above.

The new knowledge on the most contributing factors may be used in (1) clinical practice to focus on and improve the collaboration and communication between hospital physicians and GPs; focus on how to minimise premature discharges amongst older medical patients and timely sufficient post-discharge follow-up; realise the crucial roles and responsibilities of significant others to support them and promote the collaboration between healthcare professionals and significant others; focus on transitional care to bridge the gap between the hospital, GPs and home healthcare and prevent adverse events, (2) on a decision-making level to consider how to organise our healthcare systems to give all healthcare professionals the best possible conditions and resources to treat and care for the patients and (3) in in the future research to examine how to improve self-management for patients; examine how to identify and prevent relapse or worsening of medical conditions in the primary healthcare setting; examine how to sufficiently treat and care for older medical patients during the relatively short hospital admissions; and more research on how to minimise or bridge the gap in cross-sectorial patient trajectories amongst older medical patients.

In conclusion, this cross-sectional survey showed that patients, significant others and healthcare professionals perceive factors related to disease worsening, treatment and management as the most prevalent factors contributing to unplanned hospital readmission. GPs and hospital physicians as well as patients and significant others rarely agree on the contributing factors for individual patients.

## Supplementary Information

Below is the link to the electronic supplementary material.

Supplementary file1 (PDF 448 KB)

Supplementary file2 (PDF 493 KB)

Supplementary file3 (PDF 219 KB)

Supplementary file4 (PDF 237 KB)

## Data Availability

All data relevant to the study are included in the article or uploaded as supplementary information. The data cannot be shared openly. However, the anonymised data collected that supports the findings of study are available from the corresponding author (LFR), upon request.
