# Supplementary material for: Prevalence of factors contributing to unplanned hospital readmission of older medical patients when assessed by patients, their significant others and healthcare professionals: a cross-sectional survey
Source: Eur Geriatr Med. 2023 May 24;14(4):823–35. doi: 10.1007/s41999-023-00799-6 (PMC10206346; doi:10.1007/s41999-023-00799-6)
Supplement: Supplementary file 1 — Supplementary file1 (PDF 448 KB) [file 41999_2023_799_MOESM1_ESM.pdf]

Questions asked in the semi-structures interviews in the questionnaire development phase

The semi-structured interview questions were very few and open:

- *In your opinion, what contributes to readmissions among older medical patients?*
- *What comes to your mind when I say 'contributing factors to readmissions among older medical patients'?*
- *Can you describe a readmission that could have been prevented?*
- *Do you have other considerations regarding the causes of readmissions?*

Probing and clarifying questions, such as C*an you elaborate on that? Can you describe in detail…? Can you be more specific? Can you tell me about…?* and *Do you mean…?* were used when relevant.

Questionnaires for significant others and healthcare professionals

**INTROCUCTORY QUESTIONS**	
The name of the patient	
The patient's civil registration number	
Date for completing the questionnaire	
How long time has passed since the time from readmission/last consultation/attending/contact with the patient to completion of the questionnaire	☐ Less than 24 hours<br>☐ Between 1 and 3 days<br>☐ Between 3 and 5 days<br>☐ Between 5 and 7 days<br>☐ Between 7 and 14 days<br>☐ More than 14 days

DISEASE-RELATED FACTORS

To what extend do you disagree or agree that following factors could have contributed to the readmission?

Tick one box in each statement

	Strongly disagree	Disagree	Partially agree	Agree	Strongly agree	Don't know
Relapse of the condition that caused the index admission	☐	☐	☐	☐	☐	☐
The patient was not fully treated medically at discharge	☐	☐	☐	☐	☐	☐
The patient had not been diagnosed at discharge (e.g.	☐	☐	☐	☐	☐	☐

received treatment for infection and discharged without resolving the underlying cause)						
The patient could not manage symptoms or illness	☐	☐	☐	☐	☐	☐
Medication-related factors (such as side effects or incorrect dosage)	☐	☐	☐	☐	☐	☐

The patient did not show up for follow-up appointment with healthcare staff (e.g. checkups or tests) Write "not relevant" if the patient has not had any follow-up appointments since the last discharge	☐ Yes<br>☐ No<br>☐ Don’t know<br>☐ Not relevant
Worsening of other illnesses or conditions contributed to the patient's readmission. Write "not relevant" if the patient has no other illnesses or conditions	☐ Yes<br>☐ No<br>☐ Don’t know<br>☐ Not relevant
Please note here if other illness-related factors contributed to the readmission	

DIAGNOSTICS-, TREATMENT-, AND CARE- RELATED FACTORS							
To what extend do you disagree or agree that following factors could have contributed to the readmission?<br><br>Tick “not relevant” if the patient did not receive home care or if the patient has not been in contact with the GP since last dicharge<br><br>Tick one box in each statement							
	Strongly disagree	Disagree	Partially agree	Agree	Strongly agree	Don’t know	Not relevant
The patient was readmitted due to social or mental challenges	☐	☐	☐	☐	☐	☐	☐
The patient did not seek help in time	☐	☐	☐	☐	☐	☐	☐
The patient was discharged to a type of residence not suited for their care needs	☐	☐	☐	☐	☐	☐	☐
The discharge from the hospital was insufficient	☐	☐	☐	☐	☐	☐	☐
Insufficient observation or follow-up after discharge	☐	☐	☐	☐	☐	☐	☐
A new need for home care following last discharge was not met	☐	☐	☐	☐	☐	☐	☐

The patient did not receive sufficient help from home care	☐	☐	☐	☐	☐	☐	☐
Home care had difficulty managing the patient’s illness or symptoms	☐	☐	☐	☐	☐	☐	☐
Treatment via general practitioner was not initiated	☐	☐	☐	☐	☐	☐	☐
The patient's situation was too complex for the patient's medical practice to handle	☐	☐	☐	☐	☐	☐	☐
Please note here if there were other diagnostic, treatment or care related factors that contributed to the readmission							

NETWORK- RELATED FACTORS

To what extend do you disagree or agree that following factors could have contributed to the readmission?

Tick one box in each statement

	Strongly disagree	Disagree	Partially agree	Agree	Strongly agree	Don’t know

Lack of support from network (e.g. family, friends, neighbors etc.)	☐	☐	☐	☐	☐	☐
Significant others were not sufficiently included	☐	☐	☐	☐	☐	☐
Significant others wanted a readmission	☐	☐	☐	☐	☐	☐
Significant others lacked understanding of the patient's situation or plan	☐	☐	☐	☐	☐	☐
Lack of information for significant others regarding the patients' condition or plans	☐	☐	☐	☐	☐	☐
Unsatisfactory collaboration between staff and significant others	☐	☐	☐	☐	☐	☐
Please note here it there were other network related factors that contributed to the readmission						

ORGANISATION-RELATED FACTORS	
Which of the following factors contributed to the patient's readmission?<br>Tick one or more boxes	
☐ The healthcare staff was not accessible	
☐ The patient's trajectory in the healthcare system was not coherent	
☐ Lack of options for diagnostic investigation in the primary sector	
☐ Lack of options for treatment in the primary sector	
☐ Lack of alternatives to the readmission	
☐ None of the factors above contributed to the readmission	
☐ I do not know if the factors above contributed to the readmission	
Please note here if other organizational factors contributed to the readmission	

COMMUNICATION-RELATED FACTORS	
Which of the following factors contributed to the patient's readmission?<br>Tick one or more boxes	
☐ Lack of communication between healthcare professionals	
☐ Delay in the communication between healthcare professionals (e.g. a long wait for the discharge summary, care plans, correspondence etc.)	
☐ Healthcare professionals' lack of knowledge regarding treatment and care plans	
☐ Healthcare professionals did not have access to records and documentation from other institutions (e.g. the emergency service doctor did not have access to the hospital or GP records)	
☐ Plans and prescriptions were not adequately described (e.g. plans for treatment, care, rehabilitation)	
☐ Uncertainty regarding responsibility for treatment, care and follow-up	
Please note here if there were any other communication related factors that	

contributed to the readmission	

SKILLS- AND KNOWLEDGE-RELATED FACTORS	
Which of the following factors contributed to the patient's readmission?<br>Tick one or more boxes	
☐ The patient had difficulty understanding or following instructions regarding medication	
☐ The patient did not know how to seek help	
☐ The patient had difficulty understanding and following the doctors' and nurses' recommendations	
☐ The healthcare professionals lacked knowledge regarding the patient	
☐ The healthcare professionals lacked skills	
Please note here if there were other skills or knowledge related factors that contributed to the readmission	

RESOURCE-RELATED FACTORS	
Do you think that busyness and time pressure among the healthcare professionals contributed to the readmission?	☐ Yes<br>☐ No<br>☐ Don’t know
Lack of capacity in the home care (e.g. emergency beds or respite care) contributed to the readmission	☐ Yes<br>☐ No<br>☐ Don’t know
Lack of capacity at the GP (e.g. available appointments) contributed to the readmission	☐ Yes<br>☐ No<br>☐ Don’t know

Lack of capacity at the hospital (e.g. beds or available appointments in the outpatient clinics) contributed to the readmission	☐ Yes<br>☐ No<br>☐ Don’t know
Lack of room in hospice contributed to the patient's readmission<br><br>Tick “not relevant” if the patient was not in need of hospice admission	☐ Yes<br>☐ No<br>☐ Don’t know<br>☐ Not relevant
Lack of diagnostic or treatment options (e.g. blood samples, x-rays, IV treatment) contributed to the readmission	☐ Yes<br>☐ No<br>☐ Don’t know
Please note here if there were any other resource related factors that contributed to the readmission	

**PRACTICAL ARRANGEMENTS**
Which of the following factors contributed to the patient's readmission?<br>Tick one or more boxes
☐ Transportation issues for follow-up appointments
☐ Problems collecting medicine from the pharmacy
☐ The patients home was not liveable
☐ Lack of assistive aids/facilities at discharge
☐ None of the factors above contributed to the readmission
☐ I do not know if the factors above contributed to the readmission
Please note here if any other practicalities contributed to the readmission

FINAL QUESTIONS	
In your opinion, was the readmission necessary?	☐ Yes<br><br>☐ No
Were you surprised that the patient was readmitted?	☐ Yes<br><br>☐ No

Questionnaire for patients

INTROCUCTORY QUESTIONS	
Your name	
Your civil registration number	
Date for completing the questionnaire	
How long time has passed since you were readmitted (arrived to the hospital) to the time for completing this questionnaire?	☐ Less than 24 hours<br>☐ Between 1 and 3 days<br>☐ Between 3 and 5 days<br>☐ Between 5 and 7 days<br>☐ Between 7 and 14 days<br>☐ More than 14 days

DISEASE-RELATED FACTORS	
Do you think that relapse of your illness or condition contributed to your admission?	☐ Yes<br>☐ No<br>☐ Don’t know
Do you think that worsening of other illnesses or conditions contributed to your admission?<br>Write "not relevant" if you do not have other illnesses or conditions	☐ Yes<br>☐ No<br>☐ Don’t know<br>☐ Not relevant
You did not feel ready for discharge and that contributed to your admission	☐ Yes<br>☐ No<br>☐ Don’t know
You were not fully treated medically at discharge and that contributed to your readmission	☐ Yes<br>☐ No

	☐ Don’t know
You had not been diagnosed at discharge and that contributed to your readmission (e.g. you were treated for infection in your body but the cause of the infection was not found)	☐ Yes<br>☐ No<br>☐ Don’t know
You did not show up for follow-up appointment with healthcare staff and that contributed to your readmission.<br>Write "not relevant" if you did not had any follow-up appointments since the last discharge	☐ Yes<br>☐ No<br>☐ Don’t know<br>☐ Not relevant
You had difficulties managing your symptoms or illness and that contributed to your readmission.	☐ Yes<br>☐ No<br>☐ Don’t know
Side effects from medication, incorrect dosage or other drug related things contributed to your readmission.	☐ Yes<br>☐ No<br>☐ Don’t know
Please note here if you think other illness-related factors contributed to your readmission	

**DIAGNOSTICS-, TREATMENT-, AND CARE- RELATED FACTORS**	
You did not seek help in time and that contributed to your readmission	☐ Yes<br>☐ No<br>☐ Don’t know

You were discharged to a type of residence not suited for your care needs and that contributed to your readmission	☐ Yes<br>☐ No<br>☐ Don't know
There was not enough observation or follow-up after discharge and that contributed to your readmission	☐ Yes<br>☐ No<br>☐ Don't know
You had a new need for home care following last discharge which was not met and that contributed to your readmission	☐ Yes<br>☐ No<br>☐ Don't know
You did not receive sufficient help from the home care and that contributed to your readmission	☐ Yes<br>☐ No<br>☐ Don't know
Your situation and illness was too difficult for the home care to manage and that contributed to your readmission.<br>Tick "no relevant" if you do not receive home care	☐ Yes<br>☐ No<br>☐ Don't know<br>☐ Not relevant
You were in need of treatment by your GP and that contributed to your readmission.<br>Tick "no relevant" if you haven't been in contact with your GP since last discharge	☐ Yes<br>☐ No<br>☐ Don't know<br>☐ Not relevant
Your medical practice cold not manage your situation and that contributed to your readmission.<br>Tick "no relevant" if you haven't been in contact with your GP since last discharge	☐ Yes<br>☐ No<br>☐ Don't know<br>☐ Not relevant

Please note here if you think other things related to diagnostic, treatment or care contributed to your readmission	

NETWORK- RELATED FACTORS	
Lack of support from your network (e.g. family, friends, neighbors etc.) contributed to your readmission	☐ Yes<br>☐ No<br>☐ Don't know
Your significant others were not sufficiently included and that contributed to your readmission	☐ Yes<br>☐ No<br>☐ Don't know
Your significant others wanted a readmission and that contributed to your readmission	☐ Yes<br>☐ No<br>☐ Don't know
Your significant others lacked understanding of your situation or plan and that contributed to your readmission	☐ Yes<br>☐ No<br>☐ Don't know
Your significant others did not receive enough information regarding your condition or plans and that contributed to your readmission	☐ Yes<br>☐ No<br>☐ Don't know
Unsatisfactory collaboration between staff and your significant others contributed to your readmission	☐ Yes<br>☐ No<br>☐ Don't know

Please not here if other things related to your network contributed to your readmission	

ORGANISATION-RELATED FACTORS	
The healthcare staff was not accessible and that contributed to your readmission	☐ Yes<br>☐ No<br>☐ Don't know
Your trajectory in the healthcare system was not coherent and that contributed to your readmission	☐ Yes<br>☐ No<br>☐ Don't know
Please note here if other things organizational tings contributed to your readmission	

COMMUNICATION-RELATED FACTORS	
Communication between health professionals was insufficient and that contributed to your readmission	☐ Yes<br>☐ No<br>☐ Don't know
The healthcare professionals was lacking knowledge regarding your treatment and care plans and that contributed to your readmission	☐ Yes<br>☐ No<br>☐ Don't know
Please note here if there were any other communication related factors that	

contributed to your readmission	

SKILLS- AND KNOWLEDGE-RELATED FACTORS	
It was difficult to understand the instructions regarding your medication and that contributed to your readmission	☐ Yes<br>☐ No<br>☐ Don’t know
It was difficult to get help and that contributed to your readmission	☐ Yes<br>☐ No<br>☐ Don’t know
It was difficult to understand and follow the doctors' and nurses' recommendations and that contributed to your readmission	☐ Yes<br>☐ No<br>☐ Don’t know
The healthcare professionals lacked knowledge regarding you and that contributed to your readmission	☐ Yes<br>☐ No<br>☐ Don’t know
The healthcare professionals lacked skills and that contributed to your readmission	☐ Yes<br>☐ No<br>☐ Don’t know
Please note here if you thing that other things related to skills or knowledge contributed to your readmission	

RESOURCE-RELATED FACTORS	
Do you think that busyness and time pressure among the healthcare professionals contributed to your readmission?	☐ Yes<br>☐ No

	☐ Don’t know
Lack of capacity in home care (e.g. emergency beds or respite care) contributed to your readmission	☐ Yes<br>☐ No<br>☐ Don’t know
Lack of capacity at GP (e.g. available appointments) contributed to your readmission	☐ Yes<br>☐ No<br>☐ Don’t know
Lack of capacity at hospital (e.g. beds or available appointments in the outpatient clinics) contributed to your readmission	☐ Yes<br>☐ No<br>☐ Don’t know
Lack of room in hospice contributed to your readmission<br><br>Tick “not relevant” if you were not in need of a hospice stay	☐ Yes<br>☐ No<br>☐ Don’t know<br>☐ Not relevant
Lack of diagnostic or treatment options (e.g. blood samples, x-rays, IV treatment) contributed to your readmission	☐ Yes<br>☐ No<br>☐ Don’t know
Please note here if you think that other things related to resources contributed to your readmission	

**PRACTICAL ARRANGEMENTS**
Which of the following factors do you think could have contributed to your readmission?<br>Tick one or more boxes

☐ Transportation issues for follow-up appointments	
☐ Problems collecting medicine from the pharmacy	
☐ Your home was not liveable	
☐ Lack of assistive aids/facilities at discharge	
☐ None of the factors above contributed to the readmission	
☐ I do not know if the factors above contributed to the readmission	
Please note here if any other practicalities contributed to your readmission	

**FINAL QUESTIONS**	
In your opinion, was your readmission necessary?	☐ Yes<br>☐ No
Were you surprised that you were readmitted?	☐ Yes<br>☐ No
