# Supplementary material for: Prevalence of factors contributing to unplanned hospital readmission of older medical patients when assessed by patients, their significant others and healthcare professionals: a cross-sectional survey
Source: Eur Geriatr Med. 2023 May 24;14(4):823–35. doi: 10.1007/s41999-023-00799-6 (PMC10206346; doi:10.1007/s41999-023-00799-6)
Supplement: Supplementary file 2 — Supplementary file2 (PDF 493 KB) [file 41999_2023_799_MOESM2_ESM.pdf]

Patient	Patients	Significant others	GPs	District nurses	Hospital physicians
1				NP	
2					
3		PR		NNH	
4					
5				NP	
6				NP	
7			%	NP	
8			%		
9				NP	
10					
11				NP	
12			%		
13			%	NP	
14				NNH	
15			%		
16			%		
17			%		
18					
19			%		
20				NNH	
21				NP	
22				NP	
23					
24					
25			%	NP	
26					
27					
28		PR	%	NNH	
29					
30				NP	
31				NP	
32					
33					
34					
35					
36				NP	
37					
38				NP	
39		PR	%	NP	
40			%	NP	
41			%	NP	
42			%	NP	
43					
44					
45				NP	
46					
47					
48					
49					
50				NNH	
51					
52		PR			
53					
54				NP	
55				NP	
56					
57		PR		NP	

**58**				NP	
**59**			%		
**60**					
**61**					
**62**					
**63**			%	NNH	
**64**				NP	
**65**				NP	
**66**					
**67**			%	NP	
**68**			%		
**69**					
**70**					
**71**		PR			
**72**					
**73**			%	NP	
**74**				NP	
**75**		PR	%	NNH	
**76**			%		
**77**			%	NP	
**78**				NP	
**79**				NNH	
**80**					
**81**					
**82**					
**83**					
**84**				NP	
**85**			%		
**86**				NP	
**87**				NNH	
**88**			%		
**89**		PR		NP	
**90**					
**91**					
**92**			%		
**93**				NP	
**94**					
**95**				NP	
**96**					
**97**			%		
**98**			%		
**99**			%		
**100**				NNH	
**101**				NP	
**102**				NP	
**103**		PR		NP	
**104**		PR		NNH	
**105**			%	NNH	
**106**			%	NNH	
**107**				NP	
**108**			%	NP	
**109**					
**110**			%	NNH	
**111**				NNH	
**112**		PR		NNH	
**113**				NP	
**114**				NP	
**115**					
**116**				NP	
**117**				NP	

**118**		PR		NP	
**119**				NP	
**120**			%	NP	
**121**			%	NP	
**122**		PR	%		
**123**					
**124**				NP	
**125**		PR	%	NNH	
**126**				NP	
**127**					
**128**				NNH	
**129**			%	NP	
**130**				NP	
**131**			%		
**132**			%	NNH	
**133**			%		
**134**				NP	
**135**				NP	
**136**					
**137**					
**138**				NP	
**139**					
**140**				NP	
**141**			%	NP	
**142**			%	NP	
**143**			%		
**144**			%		
**145**					
**146**					
**147**				NP	
**148**					
**149**		PR		NP	
**150**				NP	
**151**				NP	
**152**				NP	
**153**			%		
**154**		PR		NNH	
**155**					
**156**			%		
**157**				NP	
**158**					
**159**				NP	
**160**			%	NP	
**161**		PR	%	NP	
**162**			%	NP	
**163**				NP	
**164**					
**165**		PR	%	NNH	
**TOTAL**	Responders<br>n= 131<br><br>Non-response<br>N=34	Responders<br>n= 130<br><br>Not included<br>n=18<br><br>Non-responders<br>N= 17	Responders<br>N= 63<br><br>Not included – no contact<br>N= 50<br><br>Non-responders<br>N=52	Responders<br>N=64<br><br>Not included<br>Horsens n=69<br><br>Not included<br>No care need<br>n=21<br><br>Non-responders<br>n=11	Responders<br>n=148<br><br>Non-responders<br>n=17

%; did not have contact with the patient between index admission and readmission, NP: home health care in Horsens municipality did not participate in the study, NNH; patient not in need of home health care, PR; relatives not participating in the study
Colour indication: grey: did not participate in the study, green participated and responded, red: non-responders who received the questionnaire but did not complete it
