# Supplementary material for: Prevalence of factors contributing to unplanned hospital readmission of older medical patients when assessed by patients, their significant others and healthcare professionals: a cross-sectional survey
Source: Eur Geriatr Med. 2023 May 24;14(4):823–35. doi: 10.1007/s41999-023-00799-6 (PMC10206346; doi:10.1007/s41999-023-00799-6)
Supplement: Supplementary file 3 — Supplementary file3 (PDF 219 KB) [file 41999_2023_799_MOESM3_ESM.pdf]

Contributing factors	Patients n= 131	Significant others n= 130	Hospital physicians n= 148	General practitioners n= 63	District nurses n= 64
	n, (%)	n, (%)	n, (%)	n, (%)	n, (%)
DISEASE-RELATED FACTORS					
Relapse of the condition that caused the index admission	104 (79.4)	94 (72.3)	85 (57.4)	48 (76.2)	38 (59.4)
The patient was not fully treated at the time of discharge from index admission	53 (40.8)	63 (48.5)	17 (11.5)	28 (44.4)	25 (39.1)
The patient was diagnostic unsettled at the time of hospital discharge	19 (14.5)	39 (30.2)	18 (12.3)	12 (19.0)	13 (20.6)
The patient could not manage symptoms or illness	48 (36.6)	59 (45.7)	51 (34.5)	25 (39.7)	29 (46.0)
Medication-related factors	17 (13.0)	18 (13.8)	11 (7.5)	4 (6.5)	6 (9.4)
The patient did not show up for follow-up appointment with healthcare staff	0 (0.0)	3 (10.0)	2 (3.2)	1 (3.7)	0 (0.0)
Worsening of other illnesses or conditions	14 (11.5)	33 (36.3)	59 (42.4)	30 (55.6)	27 (45.8)
Please note here if other illness-related factors contributed to the readmission	35 (26.7)	43 (33.1)	52 (35.1)	30 (47.6)	31 (47.7)
DIAGNOSTICS-, TREATMENT- AND CARE- RELATED FACTORS					
The patient was readmitted due to social or mental challenges	–	15 (13.5)	25 (17.0)	7 (11.3)	7 (11.7)
The patient did not seek help in time	16 (12.3)	11 (10.0)	7 (4.8)	5 (8.2)	4 (7.0)
The patient was discharged to a type of residence not suited for the care needs	6 (4.6)	19 (16.8)	12 (8.3)	9 (14.8)	7 (11.5)

The discharge from the hospital was insufficient	–	32 (26.4)	6 (4.1)	15 (24.2)	7 (11.5)
Insufficient observation or follow-up after hospital discharge	20 (15.5)	43 (35.2)	14 (9.5)	6 (9.5)	6 (9.8)
A new need for home care following last discharge was not met	10 (7.7)	9 (9.5)	8 (5.8)	3 (5.0)	3 (5.2)
The patient did not receive sufficient help from home care	11 (10.4)	16 (16.2)	9 (6.4)	4 (6.8)	6 (10.0)
Home care had difficulty managing the patients illness or symptoms	15 (14.9)	21 (21.0)	20 (14.4)	5 (8.5)	8 (13.8)
Treatment via general practitioner was not initiated	6 (9.2)	19 (19.8)	1 (0.8)	3 (5.1)	4 (7.0)
The patient's situation was too complex for the patient's medical practice to handle	3 (4.1)	43 (42.2)	30 (22.1)	28 (47.5)	15 (26.3)
Please note here if there were other diagnostic, treatment or care related factors that contributed to the readmission	24 (18.3)	47 (36.2)	25 (16.9)	16 (25.4)	22 (33.8)
NETWORK- RELATED FACTORS					
Lack of support from network	2 (1.5)	2 (1.5)	6 (4.1)	4 (6.3)	1 (1.6)
Significant others were not sufficiently included	2 (1.5)	23 (17.7)	4 (2.7)	4 (6.3)	2 (3.1)
Significant others wanted a readmission	35 (26.7)	52 (40.9)	15 (10.2)	6 (9.5)	13 (20.3)
Significant others lacked understanding of the patient's situation or plan	1 (0.8)	13 (10.0)	5 (3.4)	3 (4.8)	5 (7.9)
Lack of information for significant others regarding the patients' condition or plans	3 (2.3)	35 (26.9)	3 (2.1)	2 (3.2)	4 (6.3)
Unsatisfactory collaboration between staff and significant others	2 (1.5)	14 (10.8)	2 (1.4)	3 (4.8)	2 (3.1)
Please note here it there were other network-related factors that contributed to the readmission	17 (13.0)	31 (23.8)	5 (3.4)	2 (3.2)	11 (16.9)
ORGANISATION-RELATED FACTORS					
The healthcare staff was not accessible	8 (6.2)	5 (3.8)	3 (2.0)	1 (1.6)	1 (1.6)
The patient's trajectory in the healthcare system was not coherent	12 (9.3)	28 (21.5)	5 (3.4)	8 (12.7)	8 (12.5)

Lack of options for diagnostic investigation in the primary sector	–	6 (4.6)	5 (3.4)	4 (6.3)	12 (18.8)
Lack of options for treatment in the primary sector	–	8 (6.2)	23 (15.5)	17 (27.0)	22 (34.4)
Lack of alternatives to readmission	–	17 (13.1)	35 (23.6)	14 (22.2)	15 (23.4)
Please note here if other organizational factors contributed to the readmission	9 (6.9)	36 (27.7)	25 (16.9)	15 (23.8)	26 (40.0)
COMMUNICATION-RELATED FACTORS					
Lack of communication between healthcare professionals	11 (8.6)	15 (11.5)	7 (4.7)	6 (9.5)	5 (7.8)
Delay in the communication between healthcare professionals	–	4 (3.1)	2 (1.4)	0 (0.0)	0 (0.0)
Healthcare professionals' lack of knowledge regarding treatment and care plans	14 (10.9)	14 (10.8)	4 (2.7)	5 (7.9)	1 (1.6)
Healthcare professionals did not have access to records and documentation from other institutions	–	8 (6.2)	2 (1.4)	2 (3.2)	6 (9.4)
Plans and prescriptions were not adequately described	–	11 (8.5)	3 (2.0)	6 (9.5)	3 (4.7)
Uncertainty regarding responsibility for treatment, care and follow-up	–	24 (18.5)	9 (6.1)	7 (11.1)	5 (7.8)
Please note here if there were any other communication related factors that contributed to the readmission	11 (8.4)	19 (14.6)	12 (8.1)	6 (9.5)	12 (18.5)
SKILLS- AND KNOWLEDGE-RELATED FACTORS					
The patient had difficulty understanding or following instructions regarding medication	4 (3.1)	16 (12.3)	9 (6.1)	4 (6.3)	11 (17.2)
The patient did not know how to seek help	10 (7.6)	10 (7.7)	4 (2.7)	2 (3.2)	1 (1.6)

The patient had difficulty understanding and following the doctors' and nurses' recommendations	6 (4.6)	30 (23.1)	23 (15.5)	7 (11.1)	18 (28.1)
The healthcare professionals lacked knowledge regarding the patient	14 (10.7)	12 (9.2)	7 (4.7)	4 (6.3)	8 (12.5)
The healthcare professionals lacked skills	15 (11.5)	7 (5.4)	3 (2.0)	1 (1.6)	1 (1.6)
Please note here if there were other skills or knowledge related factors that contributed to the readmission	12 (9.1)	22 (16.9)	10 (6.7)	13 (20.6)	13 (20.0)
RESOURCE-RELATED FACTORS					
Do you think that busyness and time pressure among the healthcare professionals contributed to the readmission?	9 (6.9)	15 (11.7)	8 (5.4)	3 (4.8)	7 (10.9)
Lack of capacity in home care (e.g. emergency beds or respite care) contributed to the readmission	5 (3.8)	19 (14.6)	12 (8.2)	3 (4.8)	7 (10.9)
Lack of capacity at GP (e.g. available appointments) contributed to the readmission	3 (2.3)	10 (7.9)	1 (0.7)	0 (0.0)	2 (3.2)
Lack of capacity at the hospital	6 (4.6)	20 (15.9)	14 (9.6)	12 (19.4)	6 (9.5)
Lack of room in hospice contributed to the patient's readmission	0 (0.0)	1 (2.4)	1 (0.9)	1 (2.2)	0 (0.0)
Lack of diagnostic or treatment options (e.g. blood samples, x-rays, IV treatment) contributed to the readmission	11 (8.5)	18 (14.2)	18 (12.2)	9 (14.5)	14 (21.9)
Please note here if there were any other resource related factors that contributed to the readmission	9 (6.9)	21 (16.2)	9 (6.0)	5 (7.9)	9 (13.8)
PRACTICAL ARRANGEMENTS					
Transportation issues for follow-up appointments	1 (0.8)	3 (2.3)	1 (0.7)	1 (1.6)	1 (1.6)
Problems collecting medicine from the pharmacy	1 (0.8)	0 (0.0)	0 (0.0)	2 (3.2)	1 (1.6)
The patients home was not liveble	1 (0.8)	3 (2.3)	3 (2.0)	3 (4.8)	3 (4.7)
Lack of assistive aids/facilities at discharge	1 (0.8)	7 (5.4)	1 (0.7)	2 (3.2)	2 (3.1)

Please note here if any other practicalities contributed to the readmission	5 (3.8)	11 (8.5)	4 (2.7)	0 (0)	8 (12.3)
OTHER QUESTIONS					
Were you surprised that the patient was readmitted?	69 (53.1)	38 (29.5)	37 (25.5)	10 (16.1)	8 (12.7)
In your opinion, was the readmission necessary?	123 (94.6)	125 (96.9)	134 (91.2)	61 (98.4)	58 (95.1)
You did not feel ready to be discharged	46 (35.1)	−	−	−	−

−: the item was not included in the response groups’ questionnaire
