# Supplementary material for: Prevalence of factors contributing to unplanned hospital readmission of older medical patients when assessed by patients, their significant others and healthcare professionals: a cross-sectional survey
Source: Eur Geriatr Med. 2023 May 24;14(4):823–35. doi: 10.1007/s41999-023-00799-6 (PMC10206346; doi:10.1007/s41999-023-00799-6)
Supplement: Supplementary file 4 — Supplementary file4 (PDF 237 KB) [file 41999_2023_799_MOESM4_ESM.pdf]

Survey response time

Days	Patients: the time from readmission to completion of the questionnaire N= 131 n, (%)	Significant others: the time from readmission to completion of the questionnaire N=130 n, (%)	GPs: the time from the last consultation with the patient to completion of the questionnaire N=63 n, (%)	District nurses: the time from last patient contact to completion of the questionnaire N=64 n, (%)	Hospital physicians: the time from attending the patient to completion of the questionnaire N=148 n, (%)
< 1	13 (9.9)	0 (0)	6 (9.5)	17 (26.6)	10 (6.8)
1-3	68 (51.9)	36 (27.7)	9 (14.3)	2 (3.1)	41 (27.7)
3-5	37 (28.3)	28 (21.5)	12 (19.1)	13 (20.3)	12 (8.1)
5-7	6 (4.6)	23 (17.7)	7 (11.1)	8 (12.4)	13 (8.8)
7-14	7 (5.3)	26 (20.0)	13 (20.6)	12 (18.8)	36 (24.3)
> 14	0 (0)	17 (13.1)	16 (25.4)	12 (18.8)	36 (24.3)
Missing	0 (0)	0 (0)	0 (0)	0 (0)	0 (0)
